# Lifestyle changes associated with COVID-19 quarantine among young Saudi women: A prospective study

**DOI:** 10.1371/journal.pone.0250625

**Published:** 2021-04-29

**Authors:** Sara Al-Musharaf, Ghadeer Aljuraiban, Rania Bogis, Ruyuf Alnafisah, Madhawi Aldhwayan, Abd Tahrani

**Affiliations:** 1 Department of Community Health Sciences, College of Applied Medical Sciences, King Saud University, Riyadh, Saudi Arabia; 2 Institute of Metabolism and Systems Research, University of Birmingham, Birmingham, United Kingdom; 3 Centre for Endocrinology, Diabetes and Metabolism, Birmingham Health Partners, Birmingham, United Kingdom; 4 Department of Endocrinology and Diabetes, University Hospitals Birmingham NHS Foundation Trust, Birmingham, United Kingdom; University of Zurich, SWITZERLAND

## Abstract

**Background:**

Negative lifestyle behaviors are associated with an increased risk of adverse outcomes from coronavirus disease (COVID-19). This study aimed to assess lifestyle changes affecting weight, sleep, mental health, physical activity, and dietary habits prospectively from before COVID-19 to during lockdown.

**Methods:**

A total of 297 Saudi women, aged 19–30 years (mean age, 20.7 ± 1.4 years), were interviewed at two time points, before and during the quarantine. The data collected included anthropometrics, sociodemographic data, clinical history, food frequency questionnaire responses, Pittsburgh Sleep Quality Index scores, Global Physical Activity Questionnaire (GPAQ) responses, and Perceived Stress Scale measures. In addition, during quarantine, COVID-19 and nutrition-related information and Generalized Anxiety Disorder-7 and Patient Health Questionnaire-9 scores were collected. Multivariate multinomial logistic regression analysis was used to examine the indicators of weight gain and loss from before COVID-19 (baseline) until during lockdown.

**Results:**

Although approximately half of the participants did not report a weight change, 30% revealed weight loss and 18%, weight gain. The variables associated with increased weight gain were self-quarantine since COVID-19 started (OR: 5.17, 95% CI: 1.57–17.01, p = 0.007), age (OR: 1.53, 1.03–2.28, p = 0.04), and stress at baseline and during lockdown (OR: 1.15, 1.03–1.29, p = 0.01; OR: 1.10, 1.01–1.19, p = 0.03, respectively). The variables associated with a reduced risk of weight gain were the GPAQ score during lockdown (OR: 0.16, 0.04–0.66, p = 0.01), coffee consumption (OR: 0.36, 0.19–0.67, p = 0.01), and total sleep time (OR: 0.70, 0.51–0.97, p = 0.03).

**Conclusion:**

While most young Saudi women experienced no weight change during the COVID-19 lockdown, one-third lost weight and a significant proportion gained weight. Factors associated with weight, such as stress, sleep hours, physical activity, and coffee consumption, highlight the need to carefully consider those at risk during future circumstances that may require lockdowns. These factors could also aid in implementing policies for future lockdowns and support those most at risk of gaining weight.

## Introduction

The prevalence of obesity among adults has increased during the last decade to affect 650 million people (39%) worldwide [1]. In the Middle East, and especially in Saudi Arabia, the prevalence of metabolic diseases and obesity is strikingly high (40 and 33.7%, respectively) [2, 3], with a higher percentage of women with obesity than men [3]. A higher percentage of Saudi women of childbearing age have obesity than men [3], with more potential for weight gain and its adverse health consequences [4]. Obesity complications are well established, including cardiovascular diseases, type 2 diabetes mellitus, and some cancers [1]. However, more recently, several studies have shown that overweight and obesity are associated with increased risk of coronavirus disease (COVID-19) and adverse COVID-19 outcomes [1, 5]. This resulted in the development of guidance identifying people with obesity as a high-risk group and requiring them to strictly follow social distancing measures [6].

In addition to social distancing, more severe measures have been taken globally to slow the transmission of COVID-19, including quarantines and lockdowns. These restrictive measures have impacted people’s physical activity, working and shopping habits, dietary intake, and mental health [7, 8]. The health status of a particular population may also play a major role as physical activity, access to healthcare, and quality of the diet can differ between various countries and populations. However, the impact of the restrictive measures on weight remains unclear, with studies showing different findings around the world [9–12]. In light of the risks associated with obesity, this is an important relationship to examine, especially considering that during lockdown the availability of weight management treatments declined globally [13]. From a policy perspective, it is essential to identify those at risk of gaining weight in order to provide appropriate supportive strategies when such severe measures are applied again in the future.

Lockdown can alter nutritional habits, lifestyle behaviors, and mental health [7, 8]. Lifestyle disturbances include changes in levels of physical activity, increased daily sitting time, and altered sleep [7, 14, 15]. Also, mental health status is affected by COVID-19 and pandemic-related restrictions (prolonged isolation and social distancing), with increased anxiety, stress, and depression [8, 16], which may additionally disrupt lifestyle behaviors. Furthermore, stress generated by the pandemic situation, the presence of a threat perceived as "novel and unknown" (especially during the first phase of the epidemic), can also impact anxiety levels. Altogether, such changes, along with prolonged unstructured time, can affect human dietary behaviors and lead to weight gain during lockdown [14, 15].

In response to COVID-19, the Saudi Arabian government employed a series of restrictions on individual movement, whereby people were allowed to leave home from 06:00 to 19:00 during the partial lockdown and 06:00 to 15:00 during total lockdown, but only for documented purposes such as shopping for necessary supplies in the neighborhood. The lockdown started on 23 March and ended on 21 June 2020 [17] to limit the spread of COVID-19. We conducted a prospective study examining the impact of the COVID-19 restrictive measures on lifestyle behaviors including weight, sleep, mental health, physical activity, and dietary habits among young women.

## Materials and methods

### Study design and participants

This is a prospective cohort study; 297 participants completed an interview at 2 time points, once before the pandemic [18] and once during COVID-19 total mandatory lockdown. Ethical approval was obtained from the Institutional Review Board (IRB) of King Khalid University hospital (KKUH), Riyadh. All participants provided electronic informed consent prior to participating in the study.

Participants were healthy female students or graduates of King Saud University (KSU) aged 19–30 years. Exclusion criteria included non-Saudi nationality; previous diagnosis of sleep or psychiatric disorder, diabetes mellitus or gestational diabetes, tumors, heart or renal disease, or anemia; history of metabolic disorder; and pregnancy or lactation.

This study was part of a parent study conducted prior to COVID-19 [18]. As part of the parent study, participants were interviewed for a comprehensive questionnaire before COVID-19 (baseline), from February to April 2019 [18]. After 12 months, a follow-up was done with phone interview from April to May 2020, which coincided with the mandatory full-time lockdown period. During lockdown, questions addressed current weight, nutrition-related information, eating behavior during lockdown, depression, and anxiety, along with the same standardized questionnaires used at baseline, thus both interviews included a food frequency questionnaire (FFQ) and sleep, physical activity, and stress questionnaires.

KSU is the biggest public university in kingdom of Saudi Arabia, with more than 50,000 students. Thus it can be a reflective representative of Saudi’s young population. The average age of the study participants was 20.7 ± 1.4 years, and the majority had families with a mean monthly income of more than 10,000 SAR. These demographics characteristics are similar to other studies involving female students from other universities across Saudi Arabia [19, 20]. Al-sheikh et al. reported an average age of 20.4 ± 1.3 of 1258 female students at Princess Nora Bint Abdul Rahman University in Riyadh city. Barayan et al. reported an average age of 21.0 of 2516 of female students at Imam Abdulrahman Bin Faisal University in Dammam, with 49% declaring a family income of more than 10,000 SAR. Therefore, the authors assume that the sample in this study is representative of the age and socioeconomic status of young Saudi women.

The sample size was calculated with the assumption that at least 25% of women will make lifestyle changes, considering that the number of young Saudi women (aged 20–29) is 454,830 [21], so this study required a sample size of 255 at 95% CI and 80% power with the proportion of discordant pairs of 0.137. To account for potential nonresponse, this would target 320 subjects. The study managed to collect data on 297 women.

### Data collection

#### 1. General information

Participants were interviewed for sociodemographic information (college, major specialty, educational level, family income, and marital status) at baseline. Additional questions were asked during lockdown, including whether participants had graduated (yes/no) and their educational level. COVID-19-related information included changes in residency and family income, along with lockdown status, for which participants were divided into 3 groups: self-quarantine (people who chose to practice quarantine measures at the initial phase of the pandemic before mandatory governmental measures), partial mandatory lockdown, and total mandatory lockdown [14].

#### 2. Weight

In the clinic of the community health department at King Saud University, weight was measured at baseline using the InBody 770 body composition analyzer (InBody, Cerritos, CA, USA). Participants were asked to take off their shoes and only keep on light clothes. For the second time point, participants were asked to weigh themselves at home twice, after waking up in the morning with shoes off and wearing light clothes. We calculated mean weight and body mass index (BMI) during lockdown from the 2 reported measurements. Participants were determined to have gained or lost weight if their weight changed ≥4% from the first time point; otherwise, their weight was considered stable [22].

#### 3. Dietary data

The Saudi Food and Drug Administration’s food frequency questionnaire (SFDA FFQ), a validated questionnaire developed in the Arabic language, was used in this study for both time points; at baseline, face-to-face interviews were conducted, while during COVID-19 it was phone interviews [23]. The SFDA FFQ has both closed- and open-ended questions. The closed-ended questions consist of a list of 133 food items. For each item, 9 answering options are provided, and consumption frequency choices are stated as once a day, 2–3 times per day, 4–5 times per day, 6+ times per day, once a week, 2–4 times per week, 5–6 times per week, 1–3 times per month, and never or less than once a month. Open-ended questions collect items not previously listed, such as type of cooking fat, visible fat consumption, and consumption of salt and vitamins. The provided components are based on the Saudi food composition table (1996), McCance and Widdowson’s Composition of Foods Integrated Dataset (2015), and the concise New Zealand food composition tables 12th edition (2016) [24]. During the lockdown, information on whether participants were following any weight loss diet, number of meals and snacks, and frequency of sugary food consumption was also collected [15, 25]. There is also a question on changes in fast food eating habits during the lockdown (yes/no), and if there was a change, whether the reason was fear of COVID-19 (i.e., fear that food or packaging was contaminated by the virus) or time restrictions, or there was no change.

#### 4. Physical activity questionnaire

The official Arabic version of the Global Physical Activity Questionnaire (GPAQ) by the World Health Organization (WHO) was used in this study for both time points [26, 27]. The GPAQ comprises 16 questions grouped to capture physical activity undertaken in 3 domains: occupational physical activity, transport-related physical activity, and physical activity during discretionary or leisure time. This tool covers several physical activity components, such as the frequency and duration of 2 levels of intensity (vigorous or moderate intensity), along with a question regarding the number of hours spent in sedentary activities per day [27]. The total GPAQ score is assessed as an equivalent combination of moderately and vigorously intense physical activity, and respondents who achieve at least 600 metabolic equivalent (MET) minutes per week are considered to meet the WHO recommendation.

#### 5. Sleep index

An Arabic version of the Pittsburgh Sleep Quality Index (PSQI) was used to assess sleep quality at both time points [28]. This tool contains 19 items that evaluate sleep quality over a one-month interval. The 7 component scores include subjective sleep quality, sleep latency, sleep duration, sleep efficiency, sleep disturbance, sleeping medication, and daytime dysfunction. The score for each component ranges from 0 to 3. The final score of the components is added together, producing a range from 0 to 21; a higher total score indicates poorer sleep quality. This study draws on a study by Buysse and colleagues defining a total score of >5 as a poor sleep level and a score of ≤5 as a good sleep level [29].

#### 6. Perceived stress questionnaire

The Perceived Stress Scale (PSS-10) consists of 10 items, 6 negative and 4 positive. The PSS-10 is one of the most common scales used to research stress among different population groups. The scale measures depression, anxiety, and perception of poor health, as well as decreased satisfaction with self, job, and life in general, which are different psychosocial measures. We used an Arabic version [30] at both time points and the recent form, which asks about situations during the last month. Each item is rated on a 5-point scale from 0 (never) to 4 (very often). The higher the score, the higher the perceived stress [31]. Total scores range from 0–40, with 0–13, 14–26, and 27–40 corresponding to low, moderate, and severe stress, respectively.

#### 7. Depression scale

The Arabic version of the Patient Health Questionnaire (PHQ-9) was used to assess the severity of depression during the lockdown. It is a standard validated questionnaire used increasingly in both research and practice. The scale consists of 9 items; each item has 4 options asking how much each item has bothered the respondent over the past 2 weeks: 0, not at all; 1, several days; 2, more than half the days; and 4, nearly every day [32]. The total score for the 9 items ranges from 0 to 27, with 0–4, 5–9, 10–14, 15–19, and 20–27 indicating no, mild, moderate, moderately severe, and severe depression, respectively [32].

#### 8. Anxiety scale

We used the Generalized Anxiety Disorder scale of 7 items (GAD-7, Arabic version) to diagnose and screen for anxiety during the lockdown. Each item with response options is similar to the PHQ-9, which also depends on self-reported feelings during the previous 2 weeks. The total score of GAD-7 ranges from 0 to 21, with 0–4, 5–9, 10–14, and 15–21 indicating no, mild, moderate, and severe anxiety, respectively.

### Statistical analysis

Data were entered and analyzed using SPSS version 21. The normality of each quantitative variable was tested before analysis. Results were presented as mean ± SD for continuous variables. Categorical variables were presented as frequency (%). Statistical differences between before and during the lockdown were determined using paired sample t-test and McNemar’s test for continuous and categorical variables, respectively. The relationship between continuous variables was determined using correlation coefficients. Odds ratios (ORs) and 95% confidence interval (CI) for the ORs were obtained using multinomial regression analysis, with either weight gain or weight loss as a dependent variable to identify potential risk factors and stable weight as a reference category. Three models were used: model 1 was adjusted for age, bachelor’s degree, income and change in income, sleep, depression, and physical activity; model 2 was adjusted the same as model 1 with the addition of dietary intake; and model 3 was adjusted the same as model 2 with the addition of full lockdown effect. Significance was set at p < 0.05.

## Results

### General characteristics

A total of 297 female participants, with a mean age of 20.7 ± 1.4 years, completed the study (Table 1). During the COVID-19 pandemic, 87% of the women had no changes in family income. About half of the participants (48.1%) were self-quarantined since the initial COVID-19 spread in Saudi Arabia. The remaining participants started their quarantine when the mandatory partial and total lockdown began (42.8 and 9.1%, respectively).

**Table 1:** General characteristics of participants (n = 297).

Parameters		Mean ± SD/ N (%)
Age (years)		20.7 ± 1.4
**Sociodemographic parameters**		
Educational level	Bachelor’s degree	204 (68.7)
	Internship	41 (13.8)
	Graduate	49 (16.5)
	Master’s degree	3 (1.0)
Family Income	Less than 5000 SR	14 (4.7)
	5000–10,000 SR	44 (14.8)
	10,000–20,000 SR	114 (38.4)
	>20,000 SR	125 (42.1)
Marital status	Married	7 (2.4)
	Single	289 (97.3)
	Divorced	1 (0.3)
Women with children	No	96 (42.7)
	Yes	129 (57.3)
**COVID-19 pandemic related parameters**		
Change in family income	No	258 (86.9)
	Yes, decreased	21 (7.1)
	Yes, increased	18 (6.1)
Quarantine/lockdown status	Self-quarantine	143 (48.1)
	Partial mandatory lockdown	127 (42.8)
	Total mandatory lockdown	27 (9.1)
Following weight loss diet	No	255 (87.0)
	Yes	38 (13.0)
**Mental status parameters**		
Depression (PHQ-9 score)		8.4 ± 4.7
Depression categories	No depression	63 (21.2)
	Mild depression	128 (43.1)
	Moderate	78 (26.3)
	Moderate to severe	18 (6.1)
	Severe	10 (3.4)
Anxiety (GAD-7 score)		6.4 ± 4.2
Anxiety categories	No	108 (36.5)
	Mild	125 (42.2)
	Moderate	47 (15.9)
	Severe	16 (5.4)
**Dietary habits parameters**		
Number of main meals/day		2.1 ± 0.7
Number of snacks/day		1.9 ± 1.1
Sugary food consumption	Sometimes	135 (45.5)
	Rarely	10 (3.4)
	Never	3 (1.0)
Fast food ordering habits changed during lockdown	No	170 (57.2)
	Yes	127 (42.8)
Change in fast food eating habits	Fear of COVID-19	228 (80.9)
	Time restriction	24 (8.5)
	Not changed	30 (10.6)
	

Note: Data presented as mean ± SD for continuous and N (%) for categorical variables. SR, Saudi riyal; GAD-7, General Anxiety Disorder scale; and PHQ-9, Patient Health Questionnaire.

Fast food intake decreased by around 81% of participants due to fear of COVID-19 (i.e., fear that food or packaging would be contaminated by the virus). Changes in the intake of fat, protein, carbohydrate, and energy during the lockdown were –24.9 (10.5–39.2) g/day, –26.9 (16.6–37.1) g/day, –68.3 (30.7–105.9) g/day, and –822.5 (187.3–1457.7) kcal/day, respectively; all p-values ≤ 0.01 (Table 2).

**Table 2:** Comparison of characteristics at baseline and during lockdown.

Parameters		Before lockdown	During lockdown	P-value
		N = 297	n = 297	
BMI (kg/m^2^)		23.4 ± 5.1	23.0 ± 4.8	<0.001
Weight (kg)		58.4 ± 12.4	57.3 ± 11.8	<0.001
BMI categories	Underweight	33 (11.1)	32 (10.8)	1.00
	Normal	185 (62.3)	181 (60.6)	0.89
	Overweight	47 (15.8)	60 (20.2%)	0.03
	Obese	32 (10.8)	24 (8.1%)	0.04
PSS-10 score		19.3 ± 6.3	18.2 ± 6.0	0.003
Total sleeping time (hr/day)		5.1 ± 1.9	7.8 ± 1.9	<0.001
PSQI score		7.6 ± 2.9	6.2 ± 2.9	<0.001
Sleep quality	Good	42 (14.1)	89 (30.0)	<0.001
	Bad	255 (85.9)	208 (70.0)	
Sedentary time (min/day)		451.4 ± 242.1	484.9 ± 257.2	0.07
GPAQ score (MET-min/week)		1041.6 ± 1392.8	914.9 ± 1402.2	0.21
Recommended GPAQ ≥ 600 MET-min/week	No	156 (52.5)	176 (59.3)	0.08
	Yes	141 (47.5)	121 (40.7)	
Fat (g/day)		150.8 ± 128.0	123.6 ± 86.6	0.001
Protein (g/day)		124.4 ± 106.9	94.6 ± 51.8	<0.001
Carbohydrate (g/day)		423.4 ± 535.5	323.0 ± 264.6	<0.001
Energy (kcal/day)		3492.5 ± 5303.5	2559.0 ± 1502.8	0.01
Caffeine consumption (g/day)		11.6 ± 17	11.3 ± 19	0.87
Coffee consumption (mL/day)		143 ± 199	159.4 ± 243	0.35
Fast food consumption	No	11 (3.7)	170 (57.2)	<0.001
	Yes	286 (96.3)	127 (42.8)	

Note: Data presented as mean ± SD for continuous and N (%) for categorical variables. For continuous variables paired t-test was used and for categorical variables McNemar’s test was used. P-value < 0.05 considered significant. BMI, body mass index; PSS-10, Perceived Stress Scale; PSQI, Pittsburgh Sleep Quality Index; GPAQ, Global Physical Activity Questionnaire; MET, metabolic equivalent.

### Parameter changes at baseline and during COVID-19 pandemic

Overall, more than half the women had normal body weight before and during lockdown (62.3%), and about a quarter were overweight and/or obese at both time points, with a 0.4% decline from baseline (26.6% at baseline and 26.2% during lockdown). Among BMI categories, the proportion of participants with overweight increased from 15.8 to 20.2% (p = 0.03), while those with obesity decreased from 10.8 to 8.1% (p = 0.04).

The PSQI and PSS-10 scores significantly decreased during lockdown in comparison to baseline (Table 2). Further, when we specified total sleeping time, we found a significant decrease in the number of participants who were sleeping less than 7 hours (84.1% at baseline vs 43.8% during lockdown, p < 0.001).

### Parameter changes in weight gain and weight loss groups

Participants were divided based on their weight change into 3 groups: weight gain (n = 52, 18%), weight loss (n = 90, 30%), and weight stable (n = 155, 52%); changes in parameters before and during lockdown were assessed only in the weight gain and weight loss groups (Table 3).

**Table 3:** Comparison of weight change related parameters before and during lockdown.

Parameters		Weight Gain (N = 52)		P-value	Weight Loss (N = 90)		P-value
		Before lockdown	During lockdown		Before lockdown	During lockdown	
**Age (years)**		21.1 ± 1.6			20.6 ± 1.2		<0.05*
**Anthropometric parameters**							
**BMI (kg/m**^**2**^**)**		21.8 ± 4.8	23.8 ± 5.3	<0.001	25.4 ± 5.5	23.0 ± 4.8	<0.001
**Stress parameters**							
**PSS-10 score**		20.6 ± 6.3	19.3 ± 5.6	0.17	19.6 ± 6.5	18.5 ± 5.8	0.04
**Sleep parameters**							
**Total sleep time (hr/day)**		4.6 ± 1.9	7.6 ± 1.9	<0.001	5.0 ± 1.8	7.7 ± 1.9	<0.001
**PSQI score**		8.0 ± 3.2	6.3 ± 2.8	0.001	7.9 ± 2.7	6.8 ± 3.0	0.003
**Sleep status**	Good	8 (15.4)	11 (21.2)	0.61	7 (7.8)	20 (22.2)	0.004
	Bad	44 (84.6)	41 (78.8)		83 (92.2)	70 (77.8)	
**Physical activity parameters**							
**Sedentary time (min/day)**		414.7 ± 207.7	538.4±329.7	0.001	479.2 ± 228.5	460.4 ± 228.1	0.63
**GPAQ score**		953.1 ± 1421.1	962.3±1976.8	0.98	981.9 ± 1217.1	824.9 ± 1067.2	0.33
**Recommended GPAQ ≥ 600 MET-min/week**	No	27 (51.9)	37 (71.2)	0.04	50 (55.6)	53 (58.9)	0.74
	Yes	25 (48.1)	15 (28.8)		40 (44.4)	37 (41.1)	
**Dietary parameters**							
**Fat (g/day)**		171.0±200.0	133.1±107.8	0.13	137.5 ± 78.8	119.5 ± 94.5	0.02
**Protein (g/day)**		144.6±205.2	90.4±41.8	0.001	115.7 ± 52.9	91.7 ± 49.9	<0.001
**Carbohydrate (g/day)**		569.1± 1208.9	317.6±172.1	0.008	381.5 ± 179.9	336.7 ± 390.6	0.28
**Energy (kcal/day)**		4100±7228	2535±1451	0.009	3002 ± 13409	2614 ± 1927	0.04
**Caffeine consumption (g/day)**		12.1 ± 23.5	6.6 ± 10.8	0.11	12.6 ± 12.6	14.8 ± 22.8	0.44
**Coffee consumption (mL/day)**		105.4±161.3	153.6 ± 243.1	0.25	147.2 ± 188.3	169.8 ± 223.9	0.43
**Fast food consumption**	No	2 (5.3)	17 (44.7)	<0.001	5 (6.4)	44 (56.4)	<0.001
	Yes	36 (94.7)	21 (55.3)		73 (93.6)	34 (43.6)	

Note: P-values are obtained from dependent sample t-test and McNemar’s test. BMI, body mass index: PSS-10, Perceived Stress Scale; PSQI, Pittsburgh Sleep Quality Index; GPAQ, Global Physical Activity Questionnaire; MET, metabolic equivalent.

* P-value < 0.05 obtained from independent sample t-test between weight gain and weight loss groups.

**Fig 1:**
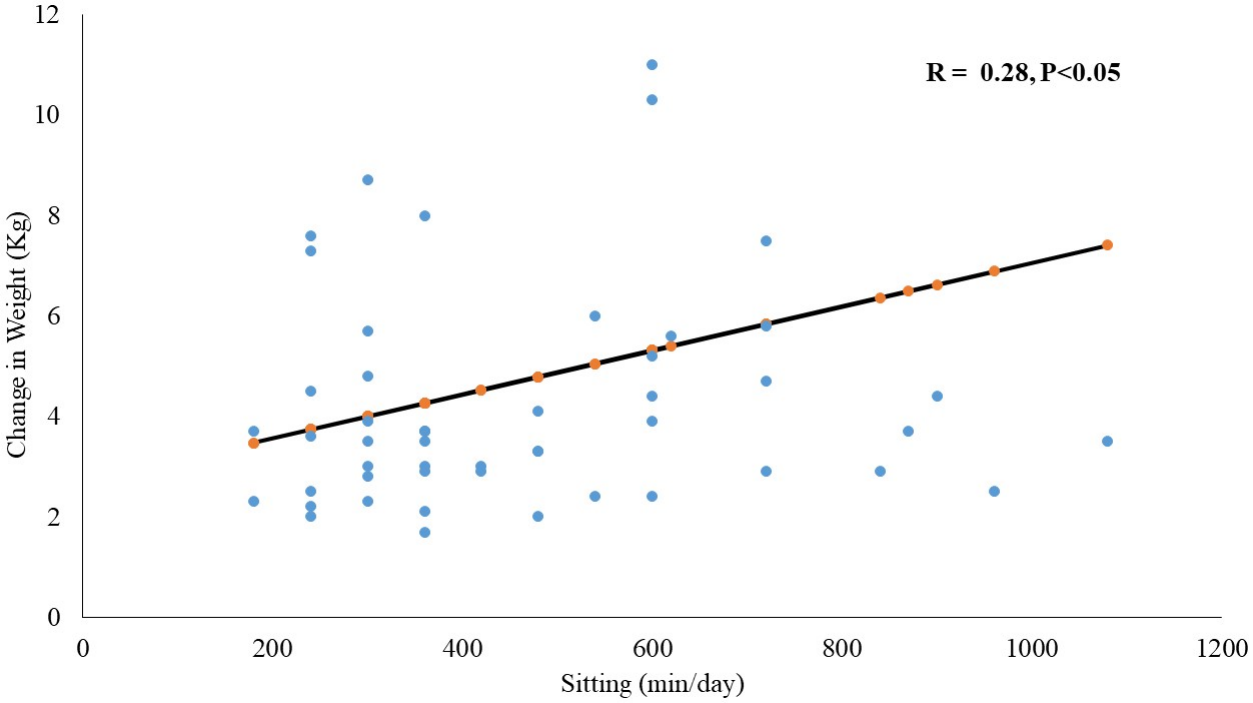
Correlation between sitting time during lockdown and weight change.

We observed that women who gained weight did not meet the recommended GPAQ score (<600 MET-min/week) during lockdown as they did before (52 vs. 71%, p = 0.04). We found a significantly positive association between weight change and sitting time (r = 0.28, p < 0.05), as shown in Fig 1.

### Predictors of weight gain and weight loss at baseline and during lockdown

Table 4 shows the results from the multivariate multinomial logistic regression analysis, highlighting the indicators of weight gain and loss. After adjustment, we found that self-quarantine since COVID-19 started (OR 5.17, 95% CI 1.57–17.01, p = 0.007), age (OR 1.53, 1.03–2.28, p = 0.04), and PSS-10 score at baseline (OR 1.15, 1.03–1.29, p = 0.01) and during lockdown (OR 1.10, 1.01–1.19, p = 0.03) were associated with weight gain.

**Table 4:** Predictors of weight gain and loss (multivariate multinomial logistic regression).

	Model 1		Model 2		Model 3	
	OR (95% CI)	P-value	OR (95% CI)	P-value	OR (95% CI)	P-value
**Weight gain**						
Age (years)	1.20 (0.92–1.57)	0.17	1.39 (0.96–2.02)	0.08	1.53 (1.03–2.28)	0.04
Educational level (bachelor’s degree or above)	0.37 (0.14–0.98)	0.04	0.38 (0.09–1.68)	0.21	0.39 (0.08–1.83)	0.23
Actual sleeping time at baseline (hr/day)	0.84 (0.65–1.07)	0.16	0.73 (0.53–1.00)	0.05	0.70 (0.51–0.97)	0.03
PSS-10 score during lockdown	1.09 (1.00–1.19)	0.06	0.98 (0.86–1.12)	0.77	0.99 (0.86–1.13)	0.87
PSS-10 score at baseline	1.05 (0.97–1.13)	0.24	1.14 (1.03–1.27)	0.01	1.15 (1.03–1.29)	0.01
GPAQ (≥ 600MET-min/week) during lockdown	0.38 (0.16–0.90)	0.03	0.16 (0.04–0.61)	0.008	0.16 (0.04–0.66)	0.01
Coffee consumption during lockdown (ml/day)	0.43 (0.23–0.77)	0.005	0.43 (0.23–0.77)	0.005	0.36 (0.19–0.67)	0.001
Self-quarantined since COVID-19 started (Yes)	2.15 (0.99–4.68)	0.053	5.17 (1.57–17.01)	0.007	5.17 (1.57–17.01)	0.007
**Weight loss**						
Number of snacks/day	0.43 (0.24–0.75)	0.003	0.43 (0.24–0.75)	0.003	0.43 (0.24–0.76)	0.004

Note: Odds ratios were obtained from multivariate multinomial logistic regression with three categories of weight change as an outcome variable and stable weight as a reference category. PSS-10, Perceived Stress Scale; GPAQ, Global Physical Activity Questionnaire; MET, metabolic equivalent. Model 1: age, bachelor’s degree, income and change in income, sleep, depression, and physical activity; model 2: model 1 + diet; model 3: model 2 + quarantine effect.

GPAQ score (≥600 MET-min/week) during lockdown (OR 0.16, 0.04–0.66, p = 0.01), coffee consumption (OR 0.36, 0.19–0.67, p = 0.01), and total sleeping time at baseline (OR 0.70, 0.51–0.97, p = 0.03) were all protective factors against weight gain, even after adjusting for confounders. The number of snacks eaten per day was protective against weight loss, even after adjusting for confounding factors (OR 0.43, 0.24–0.76, p = 0.004). Other variables were not significant.

## Discussion

This prospective study is among the first to evaluate the effect of lockdown on lifestyle factors including weight change, sleep, mental health, physical activity, and dietary habits using time points from before the pandemic to well into lockdown. The main risk factors for weight gain in this study were self-chosen quarantine during the initial spread of COVID-19 in Saudi Arabia, older age, and higher stress scores. Protective factors were meeting physical activity recommendations, coffee consumption, and increased sleeping hours.

### Weight change

Among young women, around 18% had higher weight in comparison to their pre-lockdown weight. This finding is comparable to similar studies in Italy, Spain, USA, and India during COVID-19 quarantine [9, 14, 33, 35]. These studies showed either lower weight gain, as in Spain (12%) [10], or higher weight gain, as in Poland (30–34%) [11, 12] and Chile (38%) [35]. However, these studies [9–12, 14, 33–35] relied on self-reported weight recall at the same time point and perceptions of weight rather than standardized baseline measures, and all had broad age groups and mixed genders. Thus these studies show contradicted results regarding gender and weight gain; they showed that either women gained more weight than men [23, 35, 38], or no difference [15], or men gained more weight [37]. These variations are perhaps due to different restriction levels among countries and diverse cultures and dietary patterns. We note that lockdown-associated weight gain in our study had clinical importance in the normal BMI group; this is similar to some previous studies [36, 37] and contrary to others that found weight gain mainly in the groups with obesity [12, 25, 38]. Weight gain in young women has clinical importance, as it has a tendency to substantial increase over subsequent years, with attendant comorbidities [4]. Controlling weight gain during COVID-19 and its sequelae will likely protect against general adverse health effects [1, 5].

Among the young women in our study, 30% lost weight. This result is similar to the findings in a Chinese study [37]. Other studies have reported fewer people with weight loss in comparison to ours [11, 12, 25, 33]. The weight loss reported in the present study could have been partially influenced by reduced reported fast food intake. The lockdown in Saudi Arabia and other gulf countries like Kuwait forced restaurants, malls, and delivery applications to limit their hours, decreasing fast food and restaurant meals while increasing regular home-cooked meals [39, 40]. Approximately 57% of the women in the weight-loss group decreased their total food intake and daily snacks. A Chinese study of students at two universities reported that snacking frequency was positively associated with weight gain in women only [41].

### Self-quarantine

An independent factor that increased the risk of weight gain by five times among young Saudi women was voluntary self-quarantine since the initial spread of COVID-19. Our results also show that around 50% of the sample reported usually or often eating sugary food more during than before COVID-19. This is compatible with findings in other studies showing that self-quarantine adversely affected dietary intake and habits [39, 40], increased emotional eating [42], and altered normal lifestyle behaviors [7]. This may be caused by the negative impacts of quarantine reducing the motivation to maintain healthy habits [43].

### Age

Older women showed a higher tendency to gain weight in our study. In our relatively narrow and young age group, this could be explained by women at the higher end of our range possibly having a lower metabolic rate compared to women in their early 20s [44]. Furthermore, the young age group in our study represents childbearing age [45], which may entail more stressful circumstances [46]. A study in the USA showed that adults 25 to 34 years old are less likely to follow healthy lifestyle habits and engage in more sedentary behavior than individuals 18 to 24 years old [47], and several studies have shown that weight gain is significantly associated with older adults during COVID-19 lockdown [12, 38, 48]. This observed trend is of clinical concern, since both obesity and age have been associated with a more severe course of COVID-19 and a greater risk of mortality [5].

### Stress

We found that a high risk of weight gain during lockdown was associated with moderate mental stress, whether it manifested before or arose during lockdown. This agrees with other studies showing a direct association between weight gain and increased stress due to challenging situations such as COVID-19 lockdown [41, 49]. Feeling stressed during the pandemic is expected, with women being more vulnerable than men [16–49]. Stress can be associated with food cravings and increased eating, especially among young women [42], and elevated cortisol levels that stimulate appetite, and thus weight gain [50].

### Sleep

In our study, total sleeping hours during lockdown was a protective factor against weight gain. Generally, sleeping hours significantly increased during lockdown compared to the baseline before the pandemic. However, the proportions of participants with poor sleep (70%) and short sleep of ≤7 hours during lockdown (44%) are still high. Zachary et al. found a similar association, showing a significant relationship between hours of sleep per night and reported weight gain [14]. In accordance with our findings, a meta-analysis of observational studies showed that short sleep duration was significantly associated with the risk of future obesity among adults (OR 1.41; 95% CI: 1.18–1.69) [50]. People who sleep fewer hours tend to eat more calories and macronutrients through late-night snacking [51, 52], and tend to be less motivated to eat a healthy diet [53].

### Physical activity

We found that higher GPAQ scores of >600 MET-minutes/week (the threshold recommended by the WHO) could significantly protect individuals from weight gain during lockdown [25]. Furthermore, the data revealed that during lockdown, sedentary time increased and physical activity decreased in the weight gain group (48% at baseline vs. 28.8% during lockdown). Previous studies have shown that reduced activity and increased sedentary time increase the risk of gaining weight in a dose-response manner, in general [54, 55], and especially during the COVID-19 pandemic, in both people with normal weight [11, 14, 35, 48] and with obesity [15].

In addition, several studies have shown reduced physical activity with quarantine [7, 10, 35, 38, 55]. Interestingly, two studies showed increased physical activity during quarantine [25, 56]. These differences may be related to diverse government policies on movement restrictions during the COVID-19 crisis [38].

### Dietary intake and habits

This study shows a significant reduction in macronutrient (fat, protein, and carbohydrates) as well as energy intake, which may be attributable to the significant reduction in fast food consumption during lockdown throughout the study sample. This could be related to the fear of COVID-19 transmission from either restaurant hygiene practices or delivery persons, reported by four out of five participants. Similarly, a Saudi and Kuwaiti study showed a dramatic decrease in fast food consumption during quarantine [39, 40]. However, previous findings in other countries showed that restrictions during quarantine led to increased macronutrient intake and fast food consumption [15, 25, 35], which may be due to different lockdown regulations between countries [40]. Interestingly, the increased coffee consumption in our study was found to have a protective effect against weight gain. Along with ours, other studies have reported increased coffee consumption during quarantine [40, 55]. We believe our study is one of the few to report that coffee consumption was inversely associated with weight gain. Caffeine intake could increase energy expenditure by increasing the body’s thermogenesis [57]. In animal models, caffeine suppressed increasing adiposity despite total energy intake [58].

The strengths of our study include that it was prospective, with a clearly defined time point well before and definitively during lockdown, and was conducted with a young, healthy population, while other studies have been cross-sectional or retrospective in nature [12, 14, 15]. The data collected at baseline came from in-person interviews at a clinic, with anthropometric parameters measured by the research team; data during lockdown were necessarily collected through phone call interviews. We assessed several predictors of weight changes by using internationally recognized questionnaires, including dietary intake [23], physical activity [27], sleep [28], stress [30], and anxiety and depression [59], which have been validated in Arabic versions for the Saudi population. Finally, we separately analyzed weight gain and weight loss groups within our study sample.

Our study has limitations; for example, during the second time point, lockdown prohibited taking anthropometric and blood measurements in clinic; weight was self-reported. Data relying on participant reports could be affected by recall bias and low reliability. Another limitation is that participants may have gained weight before lockdown but after the baseline data were collected. Also, we lacked some dietary factors like healthy vs. non healthy food, since our study focused more on macro- and micronutrient intake from the FFQ. Finally, the sample was made up entirely of young women from King Saud University, but as a tuition-free government university, this sample is representative of young Saudi women in general [18].

## Conclusion

In conclusion, we found some apparently protective factors against weight gain: increased actual total sleeping hours, physical activity, and coffee consumption. Interestingly, among the participants who lost weight, those with obesity lost most of the weight compared to those with normal weight. This may be attributed mainly to lower energy and overall macronutrient intake by reducing snacks and fast food during the COVID-19 lockdown. During lockdown, the need for weight management guidelines should be taken into consideration by decision makers, especially with the uncertainty around how long the pandemic is expected to last. These could include, for instance, permission to take walks and psychological support to help in reducing stress along with advice on the importance of sufficient sleeping hours to maintain a healthy lifestyle.
